# Stimulus Coding Rules for Perceptual Learning

**DOI:** 10.1371/journal.pbio.0060197

**Published:** 2008-08-12

**Authors:** Jun-Yun Zhang, Shu-Guang Kuai, Lu-Qi Xiao, Stanley A Klein, Dennis M Levi, Cong Yu

**Affiliations:** 1 State Key Laboratory of Cognitive Neuroscience and Learning, Beijing Normal University, Beijing, China; 2 School of Optometry, University of California, Berkeley, California, United States of America; 3 Helen Wills Neuroscience Institute, University of California, Berkeley, California, United States of America; Bremen University, Germany

## Abstract

Perceptual learning of visual features occurs when multiple stimuli are presented in a fixed sequence (temporal patterning), but not when they are presented in random order (roving). This points to the need for proper stimulus coding in order for learning of multiple stimuli to occur. We examined the stimulus coding rules for learning with multiple stimuli. Our results demonstrate that: (1) stimulus rhythm is necessary for temporal patterning to take effect during practice; (2) learning consolidation is subject to disruption by roving up to 4 h after each practice session; (3) importantly, after completion of temporal-patterned learning, performance is undisrupted by extended roving training; (4) roving is ineffective if each stimulus is presented for five or more consecutive trials; and (5) roving is also ineffective if each stimulus has a distinct identity. We propose that for multi-stimulus learning to occur, the brain needs to conceptually “tag” each stimulus, in order to switch attention to the appropriate perceptual template. Stimulus temporal patterning assists in tagging stimuli and switching attention through its rhythmic stimulus sequence.

## Author Summary

When a person learns to judge several stimuli in succession, like baseball pitches arriving at various speeds and spins, judgments may improve with practice only if these stimuli are presented in a fixed temporal sequence, rather than in a random order. These contrary effects suggest the need for proper stimulus coding for multi-stimulus learning in the brain. We studied how the temporal order of the stimuli affects the encoding, consolidation, and retrieval stages of perceptual learning that describe the basic stimulus coding rules throughout the learning process. We also studied why fixed stimulus sequences are required for multi-stimulus learning. Our results suggest that for multi-stimulus learning to occur, the brain needs to identify or tag each stimulus conceptually or semantically, so that the neural activity specific to each stimulus can be properly attended to. This high-level conceptual process adds to the current understanding of the mechanisms underlying perceptual learning and may have important implications for sensory training and rehabilitation.

## Abstract 3

Visual discrimination skills are more easily learned when stimuli are presented in a fixed rhythm.

## Introduction

Practice improves discrimination of fine visual features, such as contrast, orientation, vernier offset (e.g., the alignment of two lines), texture, etc. [1–6]. This process is referred to as perceptual learning and has been studied intensively in recent years because of its close links to neural plasticity [7,8] As in other forms of learning, stimulus information needs first to be encoded and consolidated into memory and later to be retrieved in order for perceptual learning to occur. Indeed, much has been done to understand stimulus coding effects on memory consolidation in a number of relevant domains. It is now clear that there are separate phases during which memory traces are susceptible to perturbation, during which they stabilize, and during which they are enhanced [9–11]. However, little is known about the coding stages of visual perceptual learning.

In a typical perceptual learning study, the observer practices a discrimination task at a single stimulus level (e.g., 30% contrast for a contrast discrimination task or 45° orientation for an orientation discrimination task), and after a few sessions of practice, discrimination at this stimulus level is usually improved. However, if the observer has to simultaneously learn discrimination of multiple stimuli (e.g., four different contrasts or orientations), perceptual learning is disabled if the stimuli are presented in a random temporal order (roving) [3,6,12,13]. In contrast, if the same stimuli are presented in a fixed temporal pattern (temporal patterning), substantial learning takes place [3].

The contrasting effects of temporal patterning and roving point to the need for proper stimulus coding to enable multiple stimulus learning. In this study we compare the effects of temporal patterning and roving and their interactions at different stages of perceptual learning, in order to reveal some basic stimulus coding principles for perceptual learning. Specifically, we investigated the roles of stimulus rhythm in perceptual learning, the effects of interruption by roving on temporal-patterned perceptual learning during consolidation and retrieval, and the minimal number of consecutive trials of the same stimulus required to escape disruption of learning through roving. Understanding the stimulus coding rules has broad implications since such multi-stimulus learning is often encountered in natural learning and is not limited to vision. Consider, for example, a baseball batter. In order to succeed, the batter needs to quickly learn to identify whether the pitch is likely to be fast or slow and whether it will curve or spin. To the best of our knowledge comparable research has not been done in other modalities, such as motor and auditory learning.

The contrasting effects of temporal patterning and roving also pose serious challenges to existing models of perceptual learning. The models based on the activities of primary visual cortex (V1) neurons, such as the model of Adini, Tsodyks, and Sagi [14,15], assume that training modifies the recurrent connections in V1 neurons, so that the local network becomes more sensitive to the trained stimulus. However, such V1-based models cannot easily explain the effects of roving and temporal patterning on multi-stimulus learning. This is because different stimuli are responded to by different sets of stimulus-tuned V1 neurons, and there is no reason to believe that training induced modification in independent local recurrent networks would be differently affected by stimulus roving and temporal patterning. Alternatively, response reweighting models, such as the Lu and Dosher model [4,16], suggest that practice improves the readout of the most relevant V1 neuron responses to the stimulus by reweighting the responses of various neurons. The response reweighting models are designed to explain single stimulus learning. In their current form they are not capable of explaining the roving and temporal patterning effects on multi-stimulus learning.

A more relevant model to our multi-stimulus learning results would be Ahissar and Hochstein's reverse hierarchy theory [17,18]. This theory proposes an easy-to-difficult stimulus learning cascade from higher to lower level brain sites, with easy stimulus learning serving as a visual attentional pointer to lower level sites for difficult stimulus learning. Stimulus temporal patterning could serve as such a pointer because it serves to “tag” the stimuli and thus improve top-down selection. Our studies elucidate the type of tagging that the reverse hierarchy theory can make use of for the top-down training of early brain regions. To deepen our understanding of the properties of tagging, in the second part of the study, we conducted a series of experiments aimed at understanding the possible mechanism underlying stimulus temporal patterning. We instantiated the findings in a stimulus tagging model for multi-stimulus perceptual learning.

## Results

### The Role of Stimulus Rhythm in Temporal Patterned Practice

With stimulus temporal patterning, multiple stimuli are presented in a fixed order with constant inter-trial intervals (ITIs) [3], so that both the stimulus sequence information and a stimulus rhythm are present. To investigate whether learning depends on stimulus sequence alone or on stimulus sequence with a rhythm, we had human observers practice multi-contrast discrimination in rhythmic and non-rhythmic sequence conditions at two ITIs. Four reference contrasts were interleaved in an ascending order (i.e., 0.2, 0.3, 0.47, and 0.63), with ITIs at 2 or 3 s (plus an observer's response time, which was more or less constant across trials), and the ITIs were either constant (2 or 3 s) or jittered (1–3 s or 2–4 s, with the mean at 2 or 3 s). The jittered ITI conditions interrupted the stimulus rhythm but preserved the stimulus sequence information.

Each experimental condition had six observers completing five 2-h practice sessions on different days, and the learning effects (Figure 1C–1F) were compared to constant 1-s fixed ITI data collected earlier [3]. Significant learning was evident in the 1-s fixed ITI condition (Figure 1B, the post/pre-training threshold ratio (PPR) = 0.69 ± 0.06; PPR < 1 indicates reduced thresholds after practice and perceptual learning). The results showed that practice with a constant 2-s ITI produced about the same amount of significant learning (PPR = 0.71 ± 0.03, Figure 1C; *F* and *p* values are presented in the figure legends) as did practice with a 1-s ITI, but increasing the constant ITI to 3 s significantly reduced learning (*p =* 0.009), although learning was still significant (PPR = 0.86 ± 0.02, Figure 1E). Jittering the ITI (for both 2 and 3 s) disabled learning (PPR = 0.99 ± 0.05 for both conditions, Figure 1D and 1F). These results suggest that for learning to occur, stimuli must be delivered in a rhythmic sequence, preferably with closer temporal proximity.

**Figure 1:**
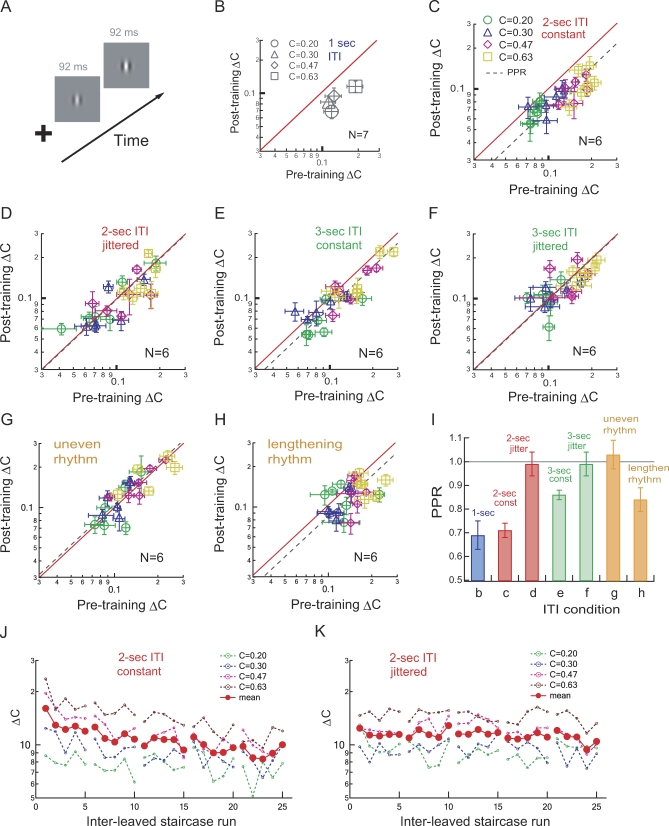
Effects of Stimulus Rhythm and ITI on Perceptual Learning of Multiple Contrast Discrimination (A) Illustration of a 2AFC trial in a contrast discrimination task. The observers judged which interval contained the higher contrast stimulus. (B) Learning effects under the 1-s constant ITI condition from our previous study [3]. In this and other plots throughout the paper, data points below the red diagonal lines indicate learning. Error bars indicate s.e.m. ΔC indicates contrast threshold. (C and D) Learning effects under 2-s constant (*F*_1,5_ = 89.9, *p <* 0.001; repeated measures ANOVA) and jittered (*F*_1,5_ = 0.08, *p =* 0.789) ITI conditions, respectively. In these and later plots, the gray dashed line indicates the mean PPR. (E and F) Learning effects under 3-s constant (*F*_1,5_ = 84.8, *p <* 0.001) (E) and jittered (*F*_1,5_ = 0.08, *p =* 0.786) ITI conditions (F). (G) Post- versus pre-training contrast thresholds with an uneven rhythm (neighboring ITIs = 2, 2.5, 1.5, and 1 s; *F*_1,5_ = 1.22, *p =* 0.320). (H) Post- versus pre-training contrast thresholds with a lengthening rhythm (neighboring ITIs = 1.25, 1.75, 2.25, and 2.75 s; *F*_1,5_ = 8.22, *p =* 0.032). (I) A summary of the learning effects in (B–H). Each bar represents the mean PPR over all four contrast conditions and all observers in the corresponding plot, as indicated along the *x*-axis. Previous result with 1-s ITI (blue colored) [3] was also plotted here for reference. (J and K) Averaged within- and between-session contrast threshold changes under 2-s constant and jittered ITI conditions (see C and D), respectively, for each reference contrast and the overall means across all reference contrasts. Each data point represents one interleaved staircase run, and each session contains five consecutive runs.

Moreover, we found that evenly spaced stimulus rhythms are most efficient for enabling perceptual learning. For the same stimulus sequence, if the rhythm was made up of uneven ITIs (i.e., 2-, 2.5-, 1.5-, and 1-s ITIs after reference contrasts of 0.2, 0.3, 0.47, and 0.63, respectively), our observers did not learn at all after 5 d of practice (PPR = 1.05 ± 0.05, Figure 1G). However, if the rhythm was more predictable (i.e., ITI lengthening from 1.25, 1.75, 2.25, to 2.75 s after reference contrasts of 0.2, 0.3, 0.47, and 0.63, respectively), learning was evident again (PPR = 0.82 ± 0.06, Figure 1H), although marginally weaker than that in the evenly spaced rhythm condition (Figure 1B; *p =* 0.105).

We also compared within- and between-session learning data under constant and jittered 2-s ITI conditions to study the dynamics of perceptual learning. Within-session learning was defined by Th(5n)/Th(5n-4), the ratio of 5th-run threshold over 1st-run threshold in the same session, where n was the session number, and between-session learning was defined by Th(5n+1)/Th(5n), the ratio of 1st-run threshold in the next session over 5th-run threshold in the current session. With rhythmic stimulus sequence (Figure 1J), the mean within-session learning index was 0.85, or on average a 15% threshold decrease within each training session. However, the between-session learning index was 1.04, suggesting no further improvement during the inter-session periods (typically 1–3 calendar days). When the stimulus rhythm was interrupted by jittered ITI (Figure 1K), the within-session learning index was 1.03 (the between-session index was 0.97), suggesting interruption of within-session learning.

### Roving Interferes with Consolidation During Perceptual Learning

The effect of trial-by-trial stimulus roving during practice suggests that the encoding of stimulus information is interrupted. However, it is unclear whether stimulus roving interferes with consolidation of perceptual learning, during which period the stimulus traces transform from working memory to long-term memory. There is evidence that consolidation of perceptual learning is mainly accomplished in the intervals between two consecutive training sessions [19–21] To examine whether consolidation is subject to roving interference, and if so for how long, we asked whether roving would disrupt learning after temporal-patterned practice.

Twenty-four observers participated in this 5-d experiment. On each day, the observers first practiced five runs of contrast discrimination, each run containing four interleaved staircases for four temporal-patterned contrasts in an ascending order (0.2, 0.3, 0.47, 0.63). They then completed one additional run of contrast discrimination with the same four contrasts, but now roving, to end that day's training. The additional roving run was delayed by 0, 4, 8, or 12 h after the end of the last temporal-patterned run. Because of the role of sleep in the consolidation of learning [21,22], there were no overnight delays, so for the 8- and 12-h delay conditions, temporal-patterned runs were done in the morning and a roving run was done in the evening.

Our previous work showed that normal temporal-patterned practice led to significant learning (mean PPR = 0.69 ± 0.06, Figure 1B and horizontal line in Figure 2E). However, the new results show that this learning was interrupted by the additional roving run 0–4 h after the temporal-patterned training. Specifically, the mean PPR over four contrasts was 0.94 ± 0.05 for the 0-h delay condition (Figure 2A), suggesting nearly completely interrupted consolidation. For the 4-h delay condition (Figure 2B) there was significant learning (mean PPR was 0.87 ± 0.04); however, it was significantly below the level with normal temporal-patterned training (*p =* 0.034). For the 8-h delay condition (Figure 2C) and the 12-h delay condition (Figure 2D), the mean PPR was 0.81 ± 0.04 and 0.78 ± 0.06, respectively. Learning with these longer delays was still below the level with normal temporal-patterned training (Figure 2E), but the difference was statistically insignificant (*p =* 0.113 when PPRs with 8-h and 12-h delay conditions combined were compared with those with normal temporal-patterned training). These results show that stimulus roving interrupts the consolidation process for at least 4 h after each practice session. After that, learning is largely consolidated and fairly immune to roving interruption.

**Figure 2:**
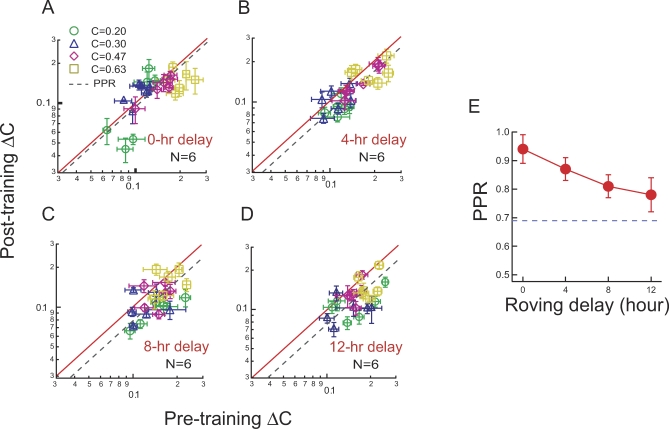
The Effects of Roving After Each Training Session on Perceptual Learning (A–D) Post- versus pre-training contrast thresholds in practice conditions in which each regular temporal-patterned training session was followed by roving interference after a delay of (A) 0 h (*F*_1,5_ = 1.48, *p =* 0.278), (B) 4 h (*F*_1,5_ = 10.6, *p =* 0.022), (C) 8 h (*F*_1,5_ = 42.0, *p =* 0.001), and (D) 12 h *(F*_1,5_ = 13.1, *p =* 0.015). (E) PPR as a function of the delay of roving interference. The horizontal line indicates the PPR in regular temporal-patterned training without followed roving interference (Figure 1B).

### The Effect of Roving on Retrieval After Completion of Learning

In the following experiments, we investigated whether roving also interrupts stimulus retrieval after successful completion of temporal-patterned training.

In the first experiment, 1 d after five sessions of temporal-patterned training, which produced significant learning (PPR = 0.71 ± 0.03, left panel of Figure 3A, replotted from Figure 1C for the same observers), six trained observers performed four roving sessions of contrast discrimination for the same reference contrasts. Contrast thresholds in the first roving session did not differ significantly from the post-training thresholds, with a mean 1st-day roving/post-training threshold ratio of 1.02 ± 0.03 (middle panel of Figure 3A). This result indicates a complete transfer of learning from the trained temporal-patterned condition to the roving condition immediately after completion of training, consistent with our earlier data [3]. Moreover, four sessions of roving runs had no significant impact on learned performance either in five observers (4th-day roving/post-training threshold ratio = 1.05 ± 0.07*,* right panel of Figure 3A the sixth observer did not complete all roving sessions). For these five observers the 4th-day roving/pre-training threshold ratio was 0.78 ± 0.07, similar to their post-/pre-training threshold ratio (0.74 ± 0.04), indicating that learning was not perturbed by extended roving interference.

**Figure 3:**
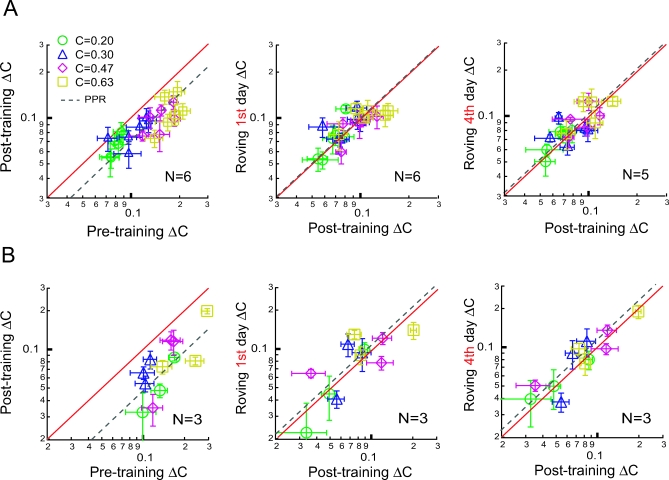
The Effects of Stimulus Roving After Learning (A) Stimulus roving immediately after completion of temporal-patterned training. The left panel shows pre- versus post-training thresholds. The middle panel shows first roving session versus post-training thresholds (*F*_1,5_ = 0.24, *p =* 0.647). Notice that the *y*-axis in the left panel becomes *x*-axis in the middle and right panels. The right panel shows the fourth (last) roving session versus post-training thresholds (*F*_1,4_ = 0.49, *p =* 0.520; only five observers finished all four roving sessions). (B) Same as (A) except that roving sessions started 2–4 wk after temporal-patterned training. The left panel shows pre- versus post-training thresholds. The middle panel shows first roving session versus post-training thresholds (*F*_1,2_ = 0.46, *p = 0.570*). Notice that the *y*-axis in the left panel becomes *x*-axis in the middle and right panels. The right panel shows the fourth (last) roving session versus post-training thresholds (*F*_1,2_ = 2.36, *p =* 0.264).

The second experiment was identical to the first, except for a 2–4-wk gap between the original temporal-patterned training and the four roving sessions. Three new observers completed this experiment. Their initial temporal-patterned training resulted in a PPR of 0.53 ± 0.06 (left panel of Figure 3B). Again there were no significant differences between post-training thresholds and first roving session thresholds (1st-day roving/post-training threshold ratio = 1.12 ± 0.14*,* middle panel of Figure 3B), or between post-training thresholds and last (fourth) roving session thresholds (post-roving/post-training threshold ratio = 1.10 ± 0.17, right panel of Figure 3B). Learning was unperturbed by four roving sessions, with a 4th-day roving/pre-training ratio of 0.58 ± 0.08. Results from these two experiments suggest that, once learned, the stimulus traces remain stable and immune to interference by multi-session roving after training; rather, they can be used to guide contrast discrimination in the roving condition.

### How Many Trials Form a Useful “Block” for Learning?

It is well documented that effective perceptual learning of multiple stimuli can occur when each stimulus (or stimulus level) is practiced in separate blocks [6,12,23]. Our working hypothesis is that when practicing discrimination with several confusable stimuli, each stimulus needs to be tagged so that the brain can attend to the appropriate perceptual template to enable learning (see next experiment and Discussion). When only a single stimulus is practiced in a block, there is no uncertainty about the stimulus tag. What is unknown is how many consecutive trials are necessary to form an effective “block,” so that stimulus traces can build up to establish the stimulus identity and resist roving disruption.

At one extreme, when multiple contrasts are roved from trial to trial, no perceptual learning occurs (Figure 4D, one-trial block size data) [6]. At the other extreme, practice in blocked trials (about 30 trials per block or staircase) produces significant learning (Figure 4D, 30-trial block size data) [6]. To determine the smallest block size for perceptual learning of multiple stimuli, we again had observers practice discrimination of four roving contrasts for five sessions, but this time each roving contrast was roved every three, five or eight consecutive trials. The mean PPR over four contrasts was 0.95 ± 0.07 for the roving-every-three-trial condition (Figure 4A and 4D), indicating that three trials was too small a block for effective learning. However, learning was evident in the roving-every-five-trial condition (PPR = 0.81 ± 0.06, Figure 4B and 4D) and became stronger in the roving-every-eight-trial condition (PPR = 0.74 ± 0.03, Figure 4C and 4D). These results suggest that a training block with as few as five consecutive trials is necessary for substantial perceptual learning of multiple stimuli with roving.

**Figure 4:**
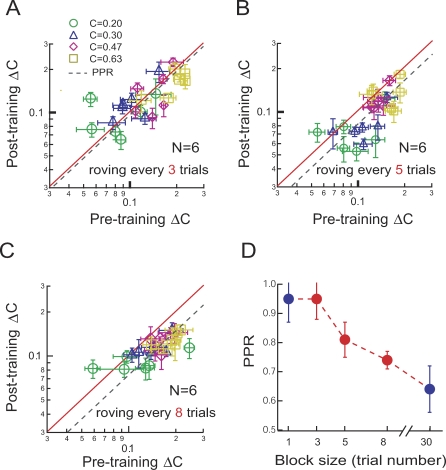
The Minimal Block Size for Perceptual Learning of Multiple Stimuli (A–C) Post- versus pre-training contrast thresholds with each roving contrast practiced (A) every three consecutive trials (*F*_1,5_ = 0.60, *p =* 0.474), (B) five consecutive trials (*F*_1,5_ = 9.26, *p =* 0.029), and (C) eight consecutive trials (*F*_1,5_ = 53.9, *p =* 0.001). (D) Summary of learning effects in various block-size conditions. Data for one-trial and 30-trial block-size conditions (blue symbols) had been reported previously [6] and were used here for reference. Each datum represents the mean PPR over four contrast conditions and all observers.

### The Role of Stimulus Identity in Perceptual Learning of Multiple-Level Stimuli

Why is perceptual learning disabled by roving but enabled by temporal patterning? One way to understand this is to seek exceptions in which perceptual learning succeeds with stimulus roving. Our initial attempts failed to find such exceptions. For example, lengthening the stimulus interval from 92 ms to 400 ms, which reduced stimulus uncertainty, produced no significant performance change (PPR = 0.91 ± 0.06, Figure 5A). Using a spatial rather than a temporal two-alternative forced choice (2AFC) paradigm, which excluded the requirement of working memory for discrimination (PPR = 1.02 ± 0.08, unpublished data), and providing a physical pre-cue identical to the reference stimulus (PPR = 0.88 ± 0.10) [3], produced no significant change either. However, we did find two cases in which roving did not disable multiple-level contrast learning.

**Figure 5:**
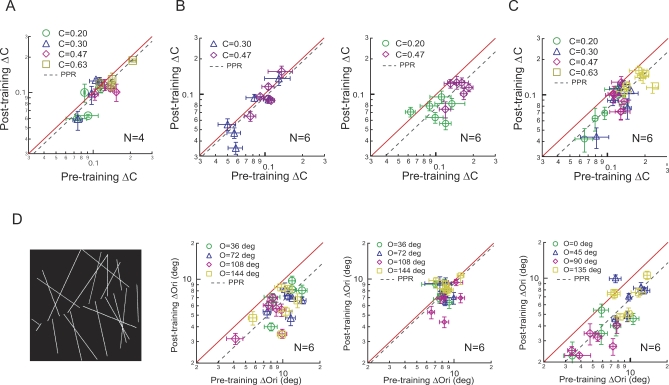
Cases in Which Learning was Undisturbed by Stimulus Roving (Except (A)) (A) Post- versus pre-training contrast thresholds for four roving contrasts with longer stimulus duration at 400 ms (*F*_1,3_ = 3.29, *p =* 0.167). (B) Post- versus pre-training contrast thresholds for (left) a more similar pair of roving contrasts (0.30 and 0.47; *F*_1,5_ = 0.79, *p =* 0.414) and (right) a more distinct pair of roving contrasts (0.20 and 0.47; *F*_1,5_ = 23.2, *p =* 0.005). (C) Post- versus pre-training contrast thresholds for four roving contrasts with pre-trial letter cues for their temporal identities (*F*_1,5_ = 34.7, *p =* 0.002). (D) Perceptual learning of orientation discrimination for illusory line stimuli (far left). Post- and pre-training orientation thresholds were compared for (middle left) the temporal patterning condition (*F*_1,5_ = 27.7, *p =* 0.003), (middle right) roving condition (*F*_1,5_ = 0.61, *p =* 0.472), and (far right) roving conditions with four cardinal or oblique orientations (*F*_1,5_ = 40.8, *p =* 0.001). Thresholds for cardinal orientations (green and purple symbols) were lower than those for oblique orientations (blue and yellow symbols), showing a classical oblique effect.

First, we had two groups of observers practice discrimination of two roving contrasts, with one group practicing the more similar pair, 0.30 and 0.47, and the other group practicing the less similar pair, 0.20 and 0.47. Perceptual learning of 0.47 contrast does not transfer to 0.30 contrast [6], so these two contrasts, though close, must be processed by independent mechanisms. After five sessions of practice, observers who practiced the more similar 0.30 and 0.47 pair showed no evidence for significant learning (PPR = 0.94 ± 0.07, left panel of Figure 5B), whereas those who practiced the less similar 0.2 and 0.47 pair showed significant learning (PPR = 0.76 ± 0.05, right panel of Figure 5B). It is interesting that roving just two close stimulus conditions was sufficient to disable perceptual learning. Similar roving effects on two stimuli have also been reported in a bisection learning task [13]. These results indicate that when the two stimuli are very different from each other (probably more than the difference of neighboring mechanisms), roving does not disrupt learning.

Second, the same four roving contrasts (0.2, 0.3, 0.47, and 0.63) were each assigned a tag—the letter A, B, C, and D, respectively. The letters provide ordinal information that could serve to identify the four roving contrasts. In each trial, the letter corresponding to one of the roving contrasts was presented on the screen 200 ms before the onset of first stimulus interval, for 200 ms. Surprisingly, letter cueing restored significant learning during roving after five sessions of training (PPR = 0.77 ± 0.04, Figure 5C). This result is all the more surprising because the letter cue is semantic, whereas previously we found that providing a direct sensory pre-cue (a Gabor patch identical to the reference Gabor) does not restore much learning [3] It appears that observers need to know the identities of the stimuli to successfully learn the roving stimuli.

To test the generality of these results, we conducted learning experiments with an illusory line orientation discrimination task (far left panel of Figure 5D) to search for conditions in which observers could learn with stimulus roving. Two groups of observers first practiced discrimination of four illusory line orientations (36°, 72°, 108°, and 144°), either with roving or with clockwise rotating temporal patterning. Significant learning was evident with orientation temporal patterning (PPR = 0.68 ± 0.06, middle left panel of Figure 5D), but was absent with orientation roving (PPR = 0.95 ± 0.06, middle right panel of Figure 5D). These data demonstrate the generality of the roles of stimulus roving and temporal patterning in perceptual learning of multiple stimuli. Moreover, unlike stimulus contrast, for which it is difficult to “know” the absolute value, some orientations, i.e. the cardinal and oblique orientations, can be judged with high confidence, and are “known” to observers and not easily confused with other orientations. Therefore, if learning multiple stimuli depends on how well the observers know the stimulus identities, as suggested in the letter cueing experiment, practice would be expected to improve discrimination of these distinct orientations even with roving. To test this notion, we had six new observers practice the four cardinal and oblique orientations with roving, and indeed we found significant learning after five sessions of training (PPR = 0.66 ± 0.05, far right panel of Figure 5D).

Taken together, these results provide important hints about the potential role of stimulus temporal patterning in multiple-stimulus learning: its sequence and rhythm assigns identities to stimuli that would otherwise be confused during learning.

## Discussion

### Stimulus Coding Rules for Perceptual Learning

Our results demonstrate that a rhythmic stimulus sequence is required to enable perceptual learning. Rhythmic stimulus presentation, especially with evenly spaced trials, would allow the observer to accurately switch attention to the outputs of the most appropriate set of neurons when the observer learns multi-level stimuli (see Figure 5 and our stimulus tagging model below). Jones et al. [24,25] proposed that attention in auditory perception is an inherently oscillatory process with adjustable periodic pulses, and that a system's responses reach maximal accuracy when attention pulses synchronize with the rhythm of the external stimuli. Our results might be consistent with an oscillation case for attention in visual perception when multi-level stimuli are being learned. Perceptual learning was significantly reduced when the ITI length changed from 2 to 3 s for the current multi-contrast discrimination task, which implies a limited length of the optimal oscillation period.

We also found that the first few hours of learning consolidation after each practice session are subject to disruption by roving. Seitz et al. [20] recently reported that consolidation after a block of training of a stimulus was interrupted if followed immediately by another block of training of a similar but different stimulus. But consolidation was little affected if the second block of training was conducted 1 h later. Although we and Seitz et al. [20] both study the effects of post-session interference on perceptual learning consolidation, there are two interesting differences. First, in Seitz et al.'s study [20], learning of one stimulus was interfered by another stimulus, whereas in our interference experiments, the stimuli were unchanged but the temporal pattern associated with the practiced stimuli, which we assume to help stimulus tagging, was interfered by a roving pattern. Second, consolidation took less time (<1 h) in Seitz et al.'s condition [20] than in ours (4 h). It is unclear whether the cognitive tagging process is responsible for extended consolidation in our results, or whether it simply takes more time to consolidate learning for multiple stimuli.

After perceptual learning has been completed, improved performance can no longer be reversed by extended training (or interference) with roving. Seitz et al. [20] reported that perceptual learning resists interference after some period of consolidation, as occurs in many learning tasks [26]. Again, the difference here is that only the temporal pattern or tagging of the stimuli is being interfered with in our study. It is likely that, once the multiple stimuli have been properly tagged after temporal-patterned training, stored stimulus information can be accurately and efficiently retrieved to guide visual discrimination regardless of the stimulus temporal context.

Our results showed that five–eight trials is the minimal block size for each roving stimulus to be learned, which, according to our model, would suggest the minimal number of trials required for stimulus tagging. An alternative explanation is that each stimulus needs to be repeated a certain number of times, so that the stimulus trace can accumulate to resist interference by the next roving stimulus, similar to Seitz et al.'s interference with consolidation by a different stimulus [20], but at a much shorter time scale (several seconds). Such stimulus trace accumulation may be facilitated by stimulus tagging in a roving situation, since newly acquired stimulus traces can now easily and correctly add to the old traces to enable learning.

### Stimulus Tagging Model

Cases in which perceptual learning escaped roving disruption (Figure 5) suggest that for multi-stimulus learning to occur, the brain needs to conceptually tag each stimulus, in order to switch attention to the appropriate perceptual template. Similar to the reverse hierarchy theory (RHT) [17,18], this proposal emphasizes top-down influence in perceptual learning, with the addition to the RHT that the top-down influence could be conceptual or semantic. The fact that direct sensory cueing (with a Gabor patch identical to the target) [3] and increased stimulus duration (Figure 5A) failed to enable learning with roving suggests that these visual cues are not sufficient to serve as effective attentional pointers for the top-down process described by the RHT [17,18]. Rather, the effect of semantic (letter) cueing on learning with roving suggests that a more conceptual process is needed to direct attention. When that conceptual cue is missing, it is difficult to achieve substantial learning. To return to our earlier example of the baseball player at bat, in order to learn quickly and efficiently to anticipate each pitch, the batter has not only to quickly recognize the subtle nuances of the pitcher's actions, and the ball's trajectory, but also to categorize the pitch appropriately as, for example, a knuckleball, curve ball, slider, or fast ball.

Stimulus temporal patterning may similarly aid learning by tagging each roving stimulus according to its “what” (sequence) and “when” (rhythm) information. Proper stimulus tagging is difficult in a roving situation because the “what” information is missing. In a recent report, perceptual learning of bisection acuity for two pairs of bisection stimuli was first interrupted by roving, but learning was later restored after extended training (ten sessions) [27]. We suspect that proper stimulus tagging was achieved with extended training in this case. A possible explanation of the roving and patterning effect is that in a 2AFC experiment, for the first interval the relevant feature of the stimulus is unknown. Thus it is difficult to attend to the appropriate filters. When there is pre-cueing or longer durations the cuing is sensory, i.e., bottom-based rather than top-down based. Our finding is that these bottom-based cues, although helpful for reducing uncertainty, are not helpful for the RHT learning. Our data show that for learning to occur, top-down attention is needed, as made possible by a semantic cue or a conceptual cue, which could be provided by the repeating rhythm. In this sense our data add to the RHT model and clarify what types of training procedures can facilitate the top-down learning.

Stimulus tagging for multi-stimulus learning can add to the current understanding of perceptual learning models. At the V1 level, the Adini, Tsodyks, and Sagi model suggests training-induced modification of recurrent connections for perceptual learning. At a post-V1 level the Lu and Dosher model suggests training-induced re-weighting of V1 neuron responses to a specific stimulus. Higher than the post-V1 level, Ahissar and Hochstein's reverse hierarchy theory proposes that visual attention serves as a top-down attentional pointer to the relevant early brain sites for perceptual learning. Now we also show that the top-down process is even affected by conceptual (semantic) processes, in that when multi-stimuli are not easily identifiable, temporal patterning or explicit identity cueing tags the stimuli. Note that our results do not argue against the lower-level models. Rather, they may together describe multi-level mechanisms operating at different stages of brain processing for effective perceptual learning.

## Methods

### Observers and apparatus.

One hundred and twenty one (121) human observers (undergraduate students at Beijing Normal University, most in their early 20s) with normal or corrected-to-normal vision participated in various phases of this study. All were new to psychophysical experiments and unaware of the specific purposes of the experiments.

The stimuli were generated by a PC-based WinVis program (Neurometrics Institute). Experiments with Gabor stimuli were run on one system using a 21-inch (about 53 cm) Sony G520 color monitor (1,024 pixel × 768 pixel, 0.37 mm (H) × 0.37 mm (V) per pixel, 120-Hz frame rate, 50 cd/m^2^ mean luminance, and 4.0° × 3.0° screen size at the 4-m viewing distance). Experiments with illusory line stimuli were run on another system using a 21-inch NEC MultiSync FE2111 color monitor (1,600 pixel × 1,200 pixel, 0.24 mm (H) × 0.24 mm (V) per pixel, 85-Hz frame rate, 41 cd/m^2^ mean luminance, and 10.67° × 8.0° screen size at the 1.5-m viewing distance). Luminance of the monitors was linearized by an 8-bit look-up table. Viewing was binocular, and a chin-and-head rest helped stabilize the heads of the observers. Experiments were run in a dimly lit room.

### Stimuli and procedure.

For contrast learning, the test stimuli were Gaussian windowed sinusoidal gratings (Gabors, Figure 1A) at a spatial frequency of 6 cycles per degree, and the standard deviation of the Gaussian envelope was 0.07°. For illusory line orientation learning, the test stimuli were ten pairs of white (full luminance) inducing lines with the inner ends aligned, which gave rise to the perception of an illusory line (the far left panel of Figure 5D). The inducing lines were 1 pixel (approximately 0.56 arcmin) wide, and their orientations were randomized for every presentation (between intervals and across trials) in the range of 30–150° from the illusory line orientation. The stimulus was presented within an invisible 4°-diameter circular black window (minimal luminance) on a uniform black screen background. The blank background was maintained through the experiment. Illusory line stimuli were viewed through a circular opening (diameter = 170°) of a black cardboard that covered the entire monitor screen. This control prevented observers from using external references to determine the orientations of the stimuli.

Contrast and orientation discrimination thresholds were measured with a temporal 2AFC staircase procedure. Staircases for all reference contrasts or orientations were run interleaved either randomly, or in an ascending (for contrasts) or clockwise (for orientations) order. For each trial (e.g., Figure 1A), the test and reference were separately presented in the two stimulus intervals (92 ms each for Gabors and 200 ms for illusory lines) in a random order separated by a 500-ms inter-stimulus interval. The observers' task was to judge which stimulus interval contained the higher contrast Gabor or more clockwise illusory line. Auditory feedback was given on incorrect responses. Each trial was preceded by a 6.3′ x 6.3′ fixation cross (300 ms) which disappeared 250 ms before the onset of the first stimulus interval. For contrast discrimination experiments, the ITI was typically 1,050 ms (ITI here included a 500-ms delay after an observer pushed a button, a 300-ms presentation of the fixation cross, and a 250-ms interval between the fixation and the onset of the first stimulus interval of the next trial. It did not count the observer's response time, which could add another few hundred milliseconds). The ITI for illusory orientation discrimination experiments was about 400 ms longer.

Each staircase consisted of two preliminary reversals and four experimental reversals. The initial contrast or orientation differences between the reference and target stimuli were sufficiently large that the observers could always make correct discrimination. The step size of the staircase was 0.05 log units. A classical three-down-one-up staircase rule was used, which resulted in a 79.4% convergence level. The geometric mean of the experimental reversals was taken as the threshold for each staircase run. When a specific staircase ended, the stimuli would still be presented at the last step value until all interleaving staircases were completed, which preserved the temporal sequence of multi-stimuli. An observer typically completed 5–6 repeats of interleaved staircase runs in a 2-h training session.

## Abbreviations

2AFC: two-alternative forced choice
ITI: inter-trial interval
PPR: post/pre-training threshold ratio
RHT: reverse hierarchy theory
